# Cardiac macrophages and emerging roles for their metabolism after myocardial infarction

**DOI:** 10.1172/JCI171953

**Published:** 2023-09-15

**Authors:** Edward B. Thorp

**Affiliations:** Department of Pathology, Feinberg School of Medicine, Northwestern University, Chicago, USA.

## Abstract

Interest in cardioimmunology has reached new heights as the experimental cardiology field works to tap the unrealized potential of immunotherapy for clinical care. Within this space is the cardiac macrophage, a key modulator of cardiac function in health and disease. After a myocardial infarction, myeloid macrophages both protect and harm the heart. To varying degrees, such outcomes are a function of myeloid ontogeny and heterogeneity, as well as functional cellular plasticity. Diversity is further shaped by the extracellular milieu, which fluctuates considerably after coronary occlusion. Ischemic limitation of nutrients constrains the metabolic potential of immune cells, and accumulating evidence supports a paradigm whereby macrophage metabolism is coupled to divergent inflammatory consequences, although experimental evidence for this in the heart is just emerging. Herein we examine the heterogeneous cardiac macrophage response following ischemic injury, with a focus on integrating putative contributions of immunometabolism and implications for therapeutically relevant cardiac injury versus cardiac repair.

## Introduction to the cardiac macrophage

In the adult, macrophages are found in most organs (1). Defined by its “professional” phagocytic prowess (2), the macrophage’s specific cellular identity is also imprinted by its organ of residence (3). In the heart, macrophage phagocytes are interwoven between the cardiac parenchyma, and account for approximately 7% of the non-cardiomyocyte cellular population (4). Thanks to single-cell technologies and elegant genetic lineage traces, we now recognize that cardiac macrophages are composed of multiple subpopulations with distinct ontological origins. These subtypes include long-lived yolk sac–derived macrophages as well as adult monocyte-derived macrophages (5, 6). Cardiac macrophages are also broadly distinguished by expression of chemokine (C-C motif) receptor 2 (CCR2), with recruited CCR2^+^ cells arising recently from circulating monocytes or adult hematopoietic lineages, and resident macrophages lacking CCR2 (CCR2**^–^**). In experimental rodents, CCR2**^–^** macrophages often arise from embryonic seeding and harbor the potential for self-renewal (5). It is important to note that this is an oversimplification (7). For example, CCR2**^–^** macrophages are further divided by expression of major histocompatibility complex (MHC) class II, as MHCII^hi^ and MHCII^lo^ populations, the latter of which are characterized as T cell immunoglobulin and mucin domain containing 4 (TimD4^+^), lymphatic vessel endothelial hyaluronan receptor 1 (Lyve1^+^), and folate receptor 2 (FolR2^+^) (7). CCR2 is also expressed in human macrophages, including monocyte-derived macrophages (8), and most human CCR2^–^ macrophages are predominantly characterized by high expression of HLA-DR^h^ (9).

Once thought to primarily act as sentinels, functioning as innate responders to blood-borne pathogens or tissue injury, cardiac macrophages are now appreciated as having more diverse functions. Various roles include those associated with critical developmental, homeostatic, and disease-associated functions. For example, during heart development, embryonic macrophages participate in coronary maturation (10) and lymphatic vessel growth, which is necessary for proper patterning of coronary blood vessels (11). In the adult, macrophages also facilitate electrical conductance by regulating the membrane potential of cardiomyocytes, and deficiency of adult macrophages induces progressive atrioventricular block (12). With aging, an increase in the contribution of blood-borne monocyte-derived macrophages to the resident pool correlates with increased inflammation (13).

## Cardiac macrophages in the acute response to myocardial infarction

Though clinical advances have reduced acute mortality after myocardial infarction (MI), ischemic heart disease remains a substantial cause of chronic morbidity and progression to heart failure (14). After infarction, macrophages are cardioprotective (15), yet also contribute to cardiac injury (16). This latter outcome is particularly relevant in the setting of reperfusion injury, where excessive inflammation portends poor clinical outcome (17). Also, blockade of CCR2-mediated recruitment of monocyte-derived macrophages after experimental MI has been shown to attenuate adverse cardiac remodeling (18). This type of injury is correlated with excess production of reactive oxygen species (ROS) (19). Moreover, aging-related defects in macrophage phagocytosis are implicated in poor cardiac outcomes (20). Detrimental functions of cardiac macrophages may be a consequence of an unevolved response to a disease that occurs in post-reproductive years.

Following MI, peripheral CCR2^+^ monocytes are recruited to the heart from the circulation and differentiate into macrophages. Monocytic accumulation follows infiltration by blood neutrophils (21), which help debride the cardiac wound but can also contribute to tissue injury. Additional signals from cardiomyocytes and myocardial B cells contribute to monocyte recruitment (22). Blood-borne macrophages serve multiple functions that protect the heart from even more devastating outcomes, such as cardiac rupture. This includes the clearance of dead parenchymal and stromal cells, which is a prerequisite for replacement fibrosis. The physical removal of actively dying cells also prevents secondary necrosis, limiting infarct expansion (23). Moreover, CCR2^+^ macrophages perpetuate inflammation by releasing monocyte chemoattractant proteins, and this fuels further monocyte recruitment (24). This positive-feedback loop is particularly important given that resident cardiac macrophage survival is markedly reduced soon after cardiac ischemia (6). Inflammatory macrophages also produce interleukin-1β (IL-1β), the blockade of which reduced clinical events in MI patients (25).

**Figure 1:**
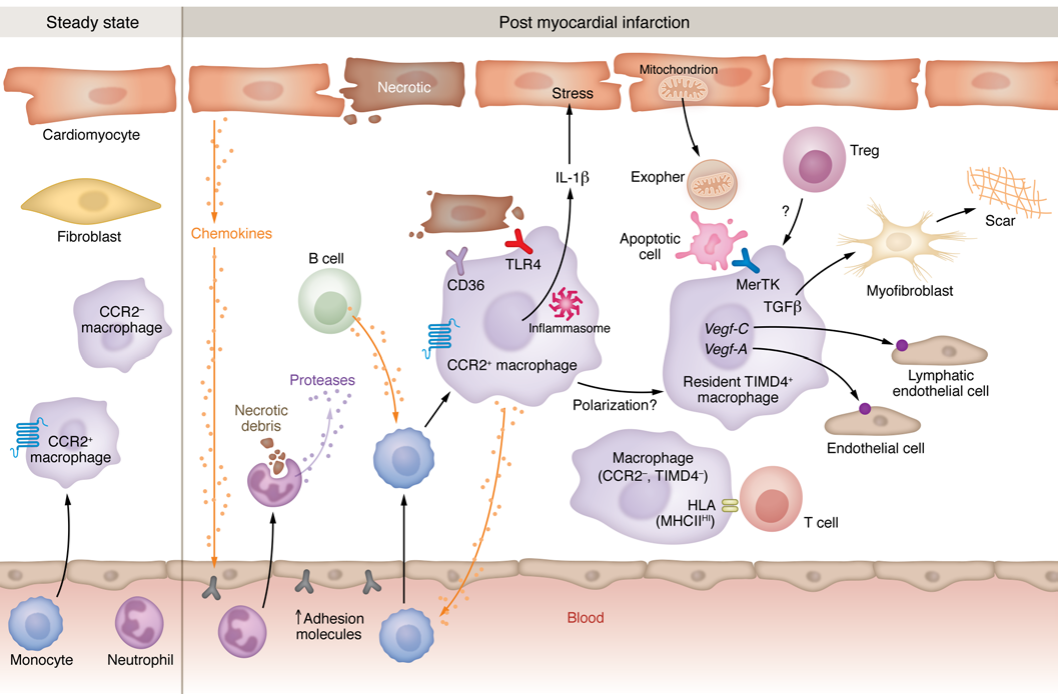
Heterogeneous cardiac macrophages and interacting cell types respond to MI. At steady state, resident CCR2**^–^** macrophages, recruited CCR2^+^ cardiac macrophages, and fibroblasts exhibit non-inflammatory activity. Within 1 week after MI, CCR2^–^ and CCR2^+^ macrophages show increased macrophage functions related to repair and inflammation. Cardiac macrophages interact with various cell types, including cardiomyocytes, neutrophils (PMNs), fibroblasts, monocytes, B cells, apoptotic cells, regulatory T cells (Tregs), endothelial cells, lymphatic endothelial cells, and myofibroblasts. Related processes include macrophage phagocytic clearance of apoptotic cells (efferocytosis) and released cardiomyocyte-derived exophers. Efferocytosis in the heart may contribute to activation of myofibroblasts and resultant scar formation. Cardiomyocytes may secrete factors that activate endothelial adhesion molecules. Macrophage receptors that interact with the cardiac milieu include the chemokine receptor CCR2, the pattern recognition receptor TLR4, and the phagocytic molecule CD36. Key effector cytokines produced after MI include IL-1β (produced from the inflammasome) and TGF-β. Reparative gene activation involves the vascular endothelial growth factors *Vegfa* and *Vegfc*.

As introduced above, cardiac macrophages are not entirely derived from monocytes, as the resident cardiac macrophage pool is prenatally seeded and maintained by self-renewal. Resident macrophages sense injury, which also triggers macrophage proliferation (26). As introduced above, and in both humans and mice, resident macrophages are classified by lack of CCR2 expression (9). In mice, CCR2**^–^** macrophages regulate monocyte fate specification, and their depletion alters the ratio of macrophage subsets following cardiac injury (24). These tissue-resident CCR2**^–^** macrophages also counterbalance inflammation through the inhibition of monocyte recruitment (24). Both mouse and human CCR2**^–^** macrophage subsets express higher levels of tissue-resident macrophage markers such as the mannose receptor CD206, the scavenger receptor CD163, the sialic acid–binding lectin SIGLEC1, and LYVE1, as well as growth factors IGF-1 and PDGF-C, relative to their CCR2^+^ counterparts (9). Thus, a dichotomy emerges whereby CCR2**^–^** cardiac macrophages are involved in orchestrating inflammation resolution and cardiac repair, in contrast to their CCR2^+^ counterparts, which act to promote inflammation (Figure 1).

## Relevance of macrophage metabolic signaling

In addition to ontogenetic factors, cell-intrinsic transcriptional and functional plasticity is a hallmark of macrophages (27). Inflammatory activation is particularly sensitive to and facilitated by triggering of macrophage Toll-like receptors (TLRs) such as TLR4 (28), the ligands of which are generated after MI (29). On the other hand, cytokines secreted from T helper 2 (Th2) lymphocytes, such as IL-4 and IL-13, polarize transcriptomes toward an antiinflammatory state (30) that includes increased expression of IL-10. In the heart, such alternatively activated macrophages appear to be critical to cardiac repair (31). Thus, macrophage polarization as a function of exposure to TLR versus IL-4 receptor ligands has been widely studied in an effort to elucidate fundamental mechanisms of functional cellular polarization. This model framework has also been exploited in vitro to investigate metabolic contributions to macrophage polarization. While this model has been tremendously insightful at the fundamental level, it is now time to prioritize a focus on in vivo mechanisms and translational potential. Our grasp of this dichotomy is particularly critical in the heart, where excessive inflammation contributes to collateral loss of non-regenerative cardiomyocytes.

Cell metabolism may conventionally be described as a balance between biosynthetic and catabolic reactions, summarized as the totality of chemical processes that are necessary to power cells via the biosynthesis of ATP. Yet a more evolved view is that metabolites also contribute to select signaling pathways that specifically regulate functional cellular plasticity. As such, it stands to reason that metabolite signaling may be sensitive to metabolite availability, which varies considerably after MI. Acute MI substantially reduces blood flow and so limits oxygen and nutrient availability within the myocardial perfusion bed that is distal to the occluded coronary artery. Access to extracellular glucose, glutamine, and lipids may also be limited, presenting a challenge to metabolically demanding macrophages.

## Inflammatory metabolism of monocytes and macrophages after acute MI

### Glucose fuels the mobilization of cardiac monocytes after MI.

Multiple lines of evidence support a paradigm wherein acute inflammatory mobilization of myeloid cells is fueled by glycolysis. Glycolysis provides a rapid pathway to generate the necessary building blocks for cell proliferation and the energy that is needed to secrete inflammatory cytokines. CCR2^+^ tissue macrophages differentiate from blood-borne monocytes, both of which increase their glucose appetite during inflammation. This thirst for sugar begins early in myeloid differentiation, external to the myocardial infarct, as disruption of glucose transporters (such as *Glut1*) in hematopoietic stem cells impairs both intracellular glucose flux and inflammatory myelopoiesis (32). In patient-derived monocytes and macrophages, increased glucose uptake fuels the generation of mitochondrial ROS (mROS). This activates the glycolytic enzyme pyruvate kinase M2 (PKM2), which in turn signals to boost IL-6 and IL-1β production. Thus, PKM2 integrates glycolysis, oxidative stress, and inflammation (33). Glycolysis is also required for the adhesion of human CD14^+^CD16^−^ monocytes to endothelial layers (34). This binding is necessary for the transmigration of blood monocytes into the heart. Many of these immunometabolic features can be recapitulated in isolated cells. For example, healthy human CD14^+^ monocytes activated with a TLR4 agonist increase aerobic Warburg (35) glycolysis and suppress mitochondrial oxidative phosphorylation (OXPHOS) (36). Glucose can also be sourced from glycogen stores to fuel inflammation (37). In patients after MI, peripheral monocytes are enriched for both glycolytic enzyme and inflammatory transcripts, and these patterns appear to be conserved between humans and experimental rodents (38). In experimental animals, monocytes just 1 day after MI exhibited higher basal extracellular acidification rates and elevated glycolytic reserve, consistent with a high glycolytic capacity. Additionally, oxygen consumption rates revealed decreased basal OXPHOS and spare respiratory capacity, and maximal respiration was also reduced, consistent with a higher dependence on glycolysis relative to oxidative metabolism (39). Glycolytic affinity in monocytes has been exploited clinically to track monocytes by fluorodeoxyglucose positron emission tomography (FDG-PET). FDG uptake is increased within the infarct concomitant with monocyte infiltration (40). In acute MI patients, peripheral monocyte abundance is also associated with elevated myocardial FDG uptake, larger infarcts, and worsened systolic function (41).

### Inflammatory and metabolic adaptations to hypoxia.

The appetite for glycolysis continues after monocytes differentiate into CCR2^+^ cardiac macrophages. Tracking macrophage polarity over the span of a week after experimental ligation of coronary arteries revealed a notable parallel induction of glycolysis and inflammatory cytokine gene transcripts (42). This response likely reflected the activity of recruited, monocyte-derived CCR2^+^ macrophages (26), which also exhibit glycolytic transcriptional signatures.

The cardiac microenvironment instructs divergent functional fates in myeloid cells during inflammation (43). Coronary obstruction reduces nutrient and oxygen (44) availability, and experimental permanent coronary occlusion leads to substantial loss of resident cardiac macrophages (6). This depleted state contrasts with that which occurs after coronary reperfusion, which salvages resident macrophages. Given that the majority of patients undergo a form of clinical reperfusion, this point stresses the importance of inducing reperfusion in studies that examine therapeutic relevance. In those cardiac macrophages that survive acute ischemia, or alternatively are recruited from the circulation, hypoxia triggers the stabilization of hypoxia-inducible transcription factors (HIFs) (45). HIF-1α promotes metabolic adaptations in a cell-autonomous manner (46), particularly by increasing mRNAs of glucose transporters and glycolytic enzymes (45). HIF-1α also polarizes toward glycolysis by suppressing metabolism through the mitochondrial tricarboxylic acid (TCA) cycle. Glycolysis is favored because HIF-1α directly transactivates the gene encoding pyruvate dehydrogenase kinase 1 (PDK1). PDK1 in turn inactivates the TCA cycle enzyme pyruvate dehydrogenase, which converts pyruvate to acetyl-CoA (47). Increased glycolytic intermediates also feed the pentose phosphate pathway (PPP), which generates NADPH-dependent ROS. Importantly, reperfusion injury has been associated with activation of NADPH oxidase in macrophages (19).

HIF-1α–dependent metabolism is further coupled to induction of inflammatory cytokines, notably IL-1β (48). Though HIFs contribute to bactericidal capacity in macrophages (49), tempering of HIF-1α activity in myeloid (50) and hematopoietic stem cells (51) has been found to attenuate inflammation and improve cardiac function after experimental MI. One immunometabolic explanation for this maladaptation is provided by shedding of cell-surface phagocytic receptors through the action of hypoxia-inducible proteases. For example, inhibiting HIF-1α and glycolysis reduced soluble levels of the phagocytic receptor MerTK, which is required for cardiac repair (52), maintenance of cardiomyocyte metabolism through removal of spent cardiomyocyte mitochondria (also known as exophers) (53), and cardiac angiogenesis (54). In addition to HIF-1α, the structurally related HIF-2α accumulates in cardiac macrophages after MI (50), and separately has been implicated in inflammatory macrophage functions (55). Like HIF-1α, HIF-2α fuels maladaptive glycolytic pathways within macrophages, albeit indirectly. That is, HIF-2α contributes to the cellular glycolytic shift by sequestering fatty acids in lipid droplets (56), in essence starving macrophage mitochondrial metabolism (50). Despite this evidence for myeloid HIF-mediated cardiopathogenic roles, others have reported that chronic hypoxia has the capacity to promote reparative macrophage functions. For example, prolonged hypoxia enhances macrophage-mediated phagocytic processes (57) through the PPP, and hypoxia preconditioning of macrophages has been shown to reduce scar size after experimental MI (58). Given the substantial interest in targeting HIFs in the setting of inflammatory ischemia/reperfusion injury (59), it will be of further interest to parse out more specific temporal and spatial contributions of hypoxic metabolism in the heterogeneous macrophage response to cardiac inflammation.

### Mitochondrial metabolism and macrophage and cardiac inflammation.

Flux of metabolites does not occur in isolation, and alterations in one pathway have consequences in another. Metabolites also enter divergent catabolic fates, depending on the activities or localization of metabolic enzymes and metabolite carriers. For example, glycolysis-derived pyruvate can be reduced to lactate in the cytosol or transported into the mitochondrion via the mitochondrial pyruvate carrier to feed oxidative metabolism. Prior studies have implicated mitochondrial pyruvate import in the inflammatory polarization of macrophages (60), although further experimentation is needed to test this concept in cardiac macrophages. Though often associated with antiinflammatory macrophage polarization, mitochondrial metabolism can also promote macrophage inflammation. For example, the mitochondrial electron transport chain is necessary for the activation of the macrophage inflammasome (61).

Within the mitochondrion of inflammatory macrophages, a so-called “broken,” or disrupted, TCA cycle explains reductions in coupled OXPHOS. This is characterized by an accumulation of TCA intermediates, including succinate, citrate, fumarate, and malate (62). In the case of succinate, its accumulation is a broad feature of ischemia and ischemic hearts (63). Upon reperfusion, accumulated succinate is oxidized by succinate dehydrogenase (SDH; complex II of the electron transport chain), leading to mROS accumulation at mitochondrial complex I (63) and in macrophages (64). mROS activates HIF-1α (65), and succinate further stabilizes HIF-1α by leading to the inhibition of prolyl hydroxylase domain (PHD) enzymes (66). Consistent with these signaling axes, inhibition of SDH reduces mROS and myocardial reperfusion-associated injury (63). For example, malonate is a competitive inhibitor of SDH and is cardioprotective when administered during cardiac ischemia (63), including by reducing infarct size (67). Succinate can further be excreted through plasma membrane transporters of the SLC13 family (68) and is recognized by neighboring cells through G protein–coupled receptor 91 (GPR91), thereby acting as a cell crosstalk signaling molecule (69). GPR91 activation in cardiomyocytes leads to pathological hypertrophy (70). Interestingly, activation of succinate receptor 1 (SUNCR1) can promote an antiinflammatory macrophage phenotype (71). This response is not completely surprising, as inflammation often programs its own resolution (72) through negative-feedback circuits.

The TCA metabolite citrate also accumulates in ischemic hearts (73). In some cases, citrate leaves the TCA cycle and mitochondrion via the citrate carrier (74). Once in the cytosol, cytosolic ATP citrate synthase, also known as ATP citrate lyase (ACLY), converts citrate to acetyl-CoA. Since this reaction increases the abundance of acetyl-CoA, which serves as a substrate for histone acetylation, it promotes alteration of the chromatin structure. Epigenetic mechanisms of inflammatory gene expression are triggered in inflammatory macrophages, and these responses correlate with increased ACLY and histone acetyltransferase activity (75). In macrophages, metabolic tracing studies revealed that TLR signaling redirects metabolic flux and generates acetyl-CoA from glucose to increase inflammatory gene expression (60). ACLY has been shown to be activated in cardiac vasculature macrophages during atherosclerosis (76), yet its role in myocardial tissue macrophages remains unknown and worth testing. The TCA intermediate fumarate is also implicated in inflammatory macrophages. Accumulation of fumarate induces monocytic epigenetic reprogramming to increase the inflammatory cytokines TNF-α and IL-6 (77). Moreover, inhibition of fumarate hydratase in macrophages leads to impaired mitochondrial respiration, the release of inflammatory mitochondrial RNA, and increased TNF production (78). Separately, knockout of fumarate hydratase in cardiomyocytes is cardioprotective after myocardial ischemia/reperfusion (79). If similar results are found after inhibition of cardiac macrophage fumarate hydratase, this approach could simplify potential therapeutic strategies; otherwise, cell-specific targeting would be necessary for optimal cardioprotection.

### Inflammatory amino acid metabolism.

Amino acids are also an important contributor to macrophage activation. For example, serine deprivation diminishes TLR4-triggered *IL1B* mRNA (80). Also, the amino acid arginine can be metabolized for either inflammatory or antiinflammatory macrophage actions. In the case of inflammation, the enzyme nitric oxide synthase (NOS) converts arginine to citrulline and nitric oxide (NO). Increased levels of inducible NOS (iNOS) are a signature feature of TLR-activated macrophages (81), and NO can antagonize mitochondrial metabolism by interfering with the activity of the TCA cycle enzyme aconitase 2 and pyruvate dehydrogenase (82). In patients, l-arginine, when added to standard postinfarction therapies, did not improve ejection fraction and may in fact may be associated with higher postinfarction mortality (83).

**Figure 2:**
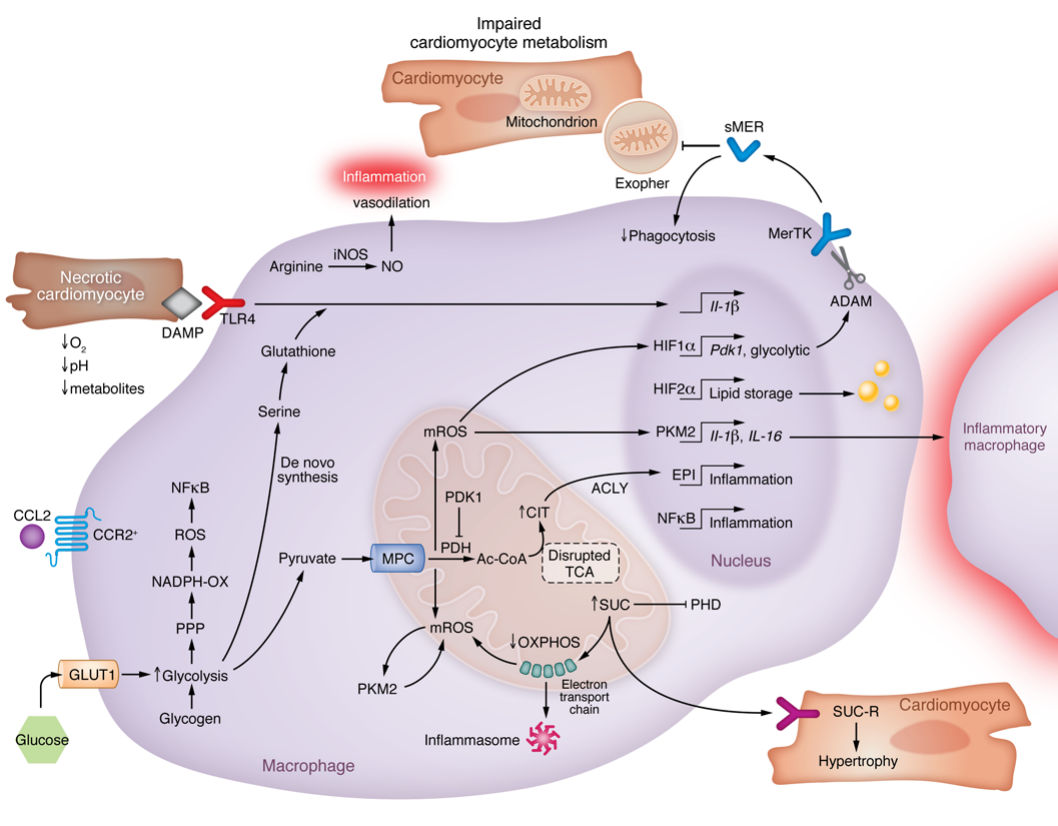
Working model of inflammatory cardiac macrophage metabolism after MI. Inflammatory immunometabolic reactions of cardiac macrophages are implicated in the response after MI. In CCR2^+^ macrophages, glucose utilization (detected by ^18^FDG-PET) through GLUT1 fuels glycolysis and inflammatory pathways including ROS, mROS, and other intermediates that promote inflammation. Metabolic HIF-1α activation may induce a disintegrin and metalloproteinase (ADAM) proteases and lead to proteolysis of phagocytic receptors such as MerTK to suppress phagocytosis of exophers and dying cells. Altered mitochondrial metabolism is also associated with the accumulation of TCA intermediates (such as succinate [SUC]) that may signal intercellular crosstalk and have the capacity to alter epigenetic regulation of proinflammatory cytokine genes. ACLY, ATP citrate lyase; CCL2, also known as monocyte chemoattractant protein-1 (MCP-1); CIT, citrate; DAMP, damage-associated molecular pattern; EPI, epigenetic; FDG, fluorodeoxyglucose; GLUT1, glucose transporter protein type 1, also known as SLC2A1; HIF-1α, hypoxia-inducible factor 1α; MPC, mitochondrial pyruvate carrier; mROS, mitochondrial reactive oxygen species; OXPHOS, oxidative phosphorylation; PDH, pyruvate dehydrogenase; PKM2, pyruvate kinase M2; PPP, pentose phosphate pathway; SDH, succinate dehydrogenase; TCA, tricarboxylic acid; TLR, Toll-like receptor.

Taken together, accumulating evidence supports a concept wherein the metabolic bias of cardiac macrophages can substantially alter the amplitude of the inflammatory response to cardiac injury. From these studies, an integrated working model emerges that illustrates potential contributions of cardiac macrophage metabolites to acute cardiac inflammation. This model is depicted in Figure 2. It is important to note that not all outcomes of these inflammatory circuits are detrimental, as inflammation per se is necessary to trigger protective inflammation-resolution and tissue repair programs. Furthermore, functions of metabolic pathways are certainly context dependent, as metabolites that act to inflame macrophages in one scenario can alternatively act in an antiinflammatory or cardiac repair capacity, as discussed below.

## Reparative cardiac macrophages

The response to cardiac injury follows a series of ordered and coordinated steps, initially relying on inflammatory macrophages examined above, followed by a macrophage functional polarization that actively dampens inflammation and orchestrates crosstalk with fibroblasts and other cell types, as examined below. As such, macrophages are dynamic and encoded with the capacity to divergently polarize over time and space (27). Tissue remodeling by macrophages requires cooperative signaling that is triggered through recognition of dying cells and the cytokine IL-4 (84). IL-4 facilitates antiinflammatory macrophage programming, which assists in preventing cardiac rupture (31). Repair macrophages also secrete TGF-β, which activates cardiac fibroblasts (85) and promotes replacement fibrosis (86). Cardiac macrophage function is also coupled to ontogeny. Embryo-derived resident cardiac macrophages exhibit enhanced tissue repair activity relative to their adult monocyte-derived counterparts (87). Unfortunately, the tissue-resident subset succumbs to cell death after prolonged ischemia (6), soon to be replenished by more inflammatory monocyte-derived macrophages. Reperfusion of coronary arteries spares the death of resident cardiac macrophages (88); however, it also amplifies inflammation by recruiting excess neutrophils and monocytes. Therefore, research into mechanisms or immunometabolites that polarize the macrophage repair response, particularly in the setting of reperfusion, is of considerable interest.

## Macrophage metabolic switches implicated in cardiac repair

Antiinflammatory macrophage polarization is often correlated with oxidative mitochondrial metabolism that is distinct from glycolysis (89). This metabolic stereotype, however, is not absolute given the integrated nature of metabolic circuitry. For example, in IL-4–treated macrophages, glutamine and glucose carbons can be traced as sources of nitrogen and carbon, respectively, into the nucleotide sugar uridine diphosphate *N*-acetylglucosamine (UDP-GlcNAc) (90), which is necessary for protein glycosylation of the prototypical antiinflammatory macrophage marker CD206. CD206, or mannose receptor, is a C-type lectin that functions in homeostatic phagocytic processes (91) and has been tested as a nuclear imaging marker for repair-phase macrophages after experimental MI (92). Glycolysis also generates lactic acid, which serves as a substrate for histone lactylation (93). Lactylation of histones can boost reparative gene activation in bone marrow and circulating monocytes, before recruitment of monocytes to infarcted hearts (39). Specifically, lactylation was enriched in the gene promoter of angiogenic *Vegfa*. Consistent with the therapeutic potential of this pathway, treatment with sodium lactate after experimental MI improved cardiac function, and this improvement was coupled to the angiogenic macrophage signature.

### Contributions of macrophage mitochondrial metabolism to repair.

After MI, macrophages increase the expression of genes that contribute to mitochondrial metabolism and OXPHOS (42). A notable mitochondrial metabolite that is induced after cardiac injury and in activated macrophages (94) is itaconate. Itaconate is generated from aconitate of the TCA cycle, and through increased expression of aconitate decarboxylase 1 (encoded by immune-responsive gene 1 [*Irg1*]), which can also be induced by TLR ligands. *Irg1* exhibits selective expression in activated immune cells (95) and inflammatory macrophages (96). Buildup of itaconate is also facilitated by reductions of isocitrate dehydrogenase 1 (*Idh1*), which in resting macrophages normally acts on aconitate in the TCA cycle to generate α-ketoglutarate (α-KG), as discussed below. Thus, itaconate is a consequence of diverting aconitate away from the TCA cycle. Itaconate has notable immunosuppressive activity (97) and can act in a negative-feedback loop during inflammation. Itaconate suppresses inflammation in part by antagonizing SDH of the TCA cycle (64). This in turn lowers mROS generation from complex I of the electron transport chain. Itaconate can also suppress inflammation by alkylating an inhibitor of the antioxidant transcription factor nuclear factor erythroid 2–related factor 2 (NRF2) (97). Moreover, itaconate has been reported to inhibit components of the inflammasome and IL-1β biosynthesis (98). These activities are not all cell intrinsic, as itaconate can also be secreted and act as an agonist on neighboring cells through targeting the G protein–coupled receptor 2-oxogluarate receptor 1 (OXGR1) (99). Thus, the therapeutic potential of itaconate has been pursued by multiple groups. Dimethyl itaconate when infused intravenously can reduce myocardial infarct size (100). Also, dimethyl itaconate delivered via subcutaneous nanofiber patches attenuated adverse remodeling after experimental MI. This effect was associated with increased antiinflammatory macrophage polarization (101). It remains unknown the degree to which itaconate is produced specifically in cardiac macrophages, and whether macrophage polarization in this setting could enhance itaconate production for therapeutic effect.

Roles for additional TCA intermediates have emerged in antiinflammatory macrophages and cardioprotection. For example, α-KG from the TCA cycle is a substrate of 2-oxoglutarate–dependent dioxygenases (2-OGDDs). A notable 2-OGDD is the HIF PHD enzyme, which acts to suppress HIF levels (102). TCA metabolites are also substrates of chromatin-modifying enzymes. For example, α-KG, fueled by glutaminolysis, contributes to alternative activation of macrophages. High ratios of α-KG to succinate favor epigenetic activation of IL-4–induced macrophage genes by histone demethylases such as Jumonji domain–containing proteins (103). Low cardiac α-KG has been implicated in myocardial ischemic injury (104), but the connection between α-KG and cardiac macrophages has not yet been made. 2-Hydroxyglutarate (2-HG) is structurally similar to α-KG, and is a metabolite that can be derived from α-KG (105). 2-HG can also rewire the epigenetic landscape by inhibition of histone and DNA demethylases (106). Exposure to TLR4 ligands leads to accumulation of 2-HG in macrophages yet appears to act in an antiinflammatory capacity. Exogenous administration of the d-2-HG enantiomer of 2-HG attenuates both macrophage TNF-α production and systemic inflammation during experimental sepsis. 2-HG protects against cardiac ischemia/reperfusion injury, yet the role of cardiac macrophages was not investigated (107).

### NAD^+^, macrophage function, and cardiac inflammation.

A key metabolic function of the TCA cycle is to generate the reducing equivalents NADH^2^ and FADH^2^. NADH is oxidized by electron transport chain complex I for OXPHOS, and this generates NAD^+^. Mitochondrial ratios of NAD^+^ to NADH are reflected to an extent in the cytosol, and this distribution occurs by transfer of reducing equivalents through mitochondrial matrix shuttles, such as the malate aspartate shuttle. Increases in cytosolic NAD^+^ cofactors can in turn activate NAD^+^-dependent signaling proteins and therefore influence cell-fate decisions. Importantly, declining NAD^+^ levels are correlated with many diseases of aging. Macrophages from aged subjects showed increased activities of the NAD^+^-consuming enzyme CD38, which is linked to macrophage inflammatory polarization (108). Reduced NAD^+^ levels are also found in experimental models of heart failure (109). Administration of NAD^+^ precursors, such as nicotinamide riboside, boost low mitochondrial respiration capacity found in peripheral blood mononuclear cells from patients with systolic heart failure (110). Moreover, NAD^+^ has been shown to restore α-KG levels, favoring HIF-1α degradation (111) and promotion of antiinflammatory macrophage polarization (103). NAD^+^ is also increased after macrophages engulf apoptotic cells (discussed more below) and is required for the biosynthesis of antiinflammatory IL-10 (112). Interestingly, exogenous supplemental NAD^+^ was found to protect myocardium against myocardial ischemic/reperfusion injury in an animal model (113).

### Amino acids, macrophage repair functions, and the heart.

Amino acid availability is critical to macrophages, which encode sensors such as mTORC1 for amino acid sensing (114). Glutamine is the most abundant amino acid in plasma and is internalized in macrophages via the solute carrier family 1 member 5 (SLC1A5) transporter. Catabolism of glutamine by glutaminolysis can contribute to inflammatory macrophage activation (such as through anaplerosis), but glutamine can also support IL-4–induced macrophage polarization (90). Glutamine is uniquely metabolized during macrophage phagocytosis of apoptotic cells (efferocytosis). This buffers the mitochondrial oxidative stress that is generated during efferocytosis (115). Also, environments enriched in glutamate fuel glutaminolysis to maintain phagocytic respiratory bursts (116). Compellingly, glutamine has been reported to improve cardiac function following experimental myocardial ischemia/reperfusion (117).

Another amino acid implicated in both reparative macrophages and the heart is arginine, the catabolism of which is intricately linked to divergent macrophage polarization phenotypes. Arginase, which hydrolyzes arginine to ornithine and urea, is induced in macrophages exposed to IL-4. During efferocytosis, metabolism of apoptotic cell–derived arginine to putrescine facilitates actin-signaling pathways that enhance multiple rounds of efferocytic engulfment (118). More recent data suggest that the import of arginine-derived polyamines (such as spermidine and spermine), also from engulfed apoptotic cells, can suppress IL-1β or IL-6 (119). Despite these inflammation resolution phenotypes, it is important to remember that in macrophages arginine can also be metabolized to inflammatory NO, as discussed above, and treatment with l-arginine is not recommended following MI (83). Thus, enhancing specific downstream metabolites of arginine may more specifically promote pathways that resolve excess inflammation.

### Metabolism of dying cells: contributions to cardiac repair.

Resolution of inflammation acts in part through the programmed cell death of accumulated immune cells. A feature of this program is to recruit macrophages, which engulf dying cells through the process of efferocytosis. If this process is inefficient, secondary necrosis can ensue and expose self-antigen to conditions that facilitate autoimmunity. For example, MI triggers chronic cardiac autoimmunity in type 1 diabetic patients (120). Separately, diabetes is a risk factor for defective efferocytosis (121), and blockade of CCR2^+^ macrophages after MI in experimental nonobese diabetic animals reduced the formation of autoimmune markers (122). In addition to physically removing dead cells, efferocytosis is also a cue to activate repair signaling from within cardiac macrophages (52). After MI, this repair process can include the expression of angiogenic (54) and lymphangiogenic factors (123). Thus, efferocytosis and dying cell metabolism by cardiac macrophages is at the interface of cardiac inflammation and cardiac repair.

After MI, a sizable mass of injured tissue must be cleared and metabolized. This includes necrotic cardiomyocytes, apoptotic bodies from apoptotic cardiomyocytes (124), and apoptotic neutrophils, which turn over rapidly at injured sites. Engulfment of dying cells is a metabolic strain for macrophages, leading to oxidative stress (125). In response, phagocytes induce antioxidant mRNAs (126). Exposure to high amounts of dying cells can lead to additional compensatory mechanisms, including the activation of both mitochondrial uncoupling proteins (127) and mitochondrial fission machinery (128). Efferocytosis also triggers increased glycolysis and generation of lactate. Secreted lactate in this context is recognized by receptors on neighboring macrophages and can promote antiinflammatory macrophage polarization (129). After MI, lactate has been shown to preserve ventricular function (130).

What is the fate of metabolites sourced from dying cells in the heart? If metabolites are limiting in ischemic tissue, it could be advantageous for cardiac macrophages to recycle or metabolize substrates from dying cells, for the purpose of tissue repair. Alternatively, engulfed metabolites could serve as signaling cues to modulate the macrophage response. Indeed, there is experimental evidence supporting these concepts, such as with arginine, as aforementioned. Moreover, apoptotic cell–derived nucleotides trigger intracellular signaling that promotes macrophage proliferation (131). This finding is particularly intriguing in light of cardiac macrophage populations that expand through proliferation (132). Thus, specific apoptotic cell–derived factors may be utilized to potentially expand selective cardioprotective macrophage populations.

Dying cells also deliver a large payload of lipids and membrane-derived cholesterol to phagocytes. While macrophage lipid accumulation is linked to inflammatory responses, such as during atherosclerosis, the process of efferocytosis triggers compensatory reactions that not only mitigate lipid-induced cellular stress (133) but also trigger tissue-reparative signaling. For example, lysosomal acid lipase hydrolyzes cholesterol esters in endocytic compartments, and this response can lead to antiinflammatory oxysterol production (134). Also, increased fatty acids in apoptotic cells contribute to antiinflammatory macrophage responses (e.g., IL-10) (112), and separately, fatty acid oxidation and activation of PPARγ coactivator-1β (PGC-1β) have been shown to polarize macrophages to an antiinflammatory phenotype (135). Furthermore, genetic silencing of mitochondrial lipid importers and components of the electron transport chain inhibit IL-10 production during efferocytosis, suggesting a role for fatty acid oxidation (112). Finally, disruption of electron transport chain complexes I (136) and III (112) in myeloid cells leads to poor outcomes after experimental MI.

**Figure 3:**
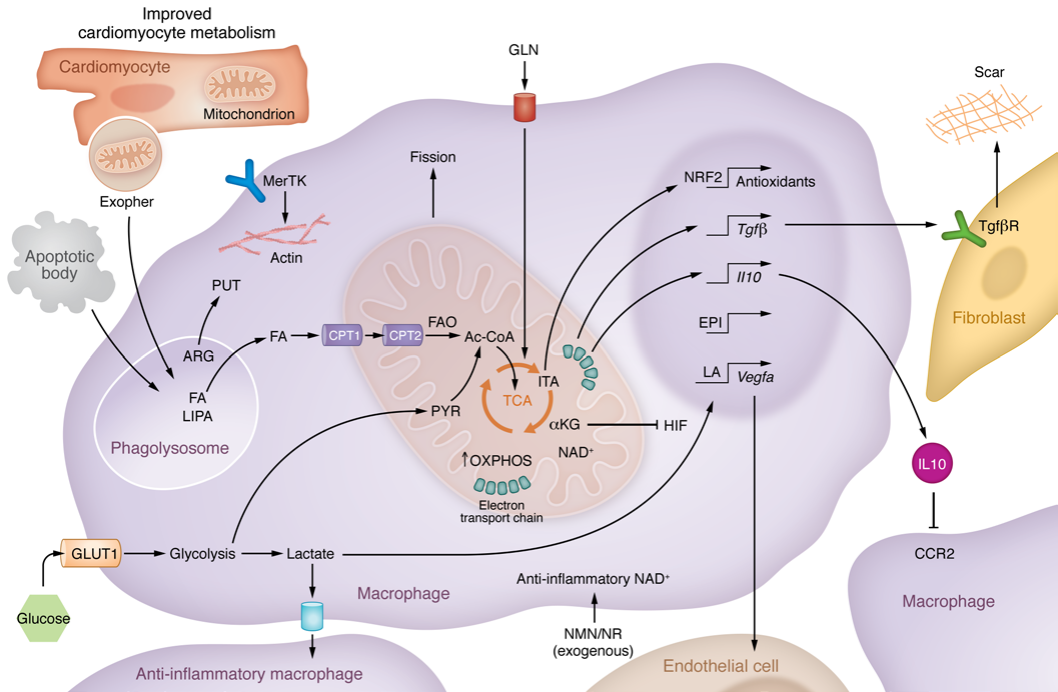
Working model of the contributions of cardiac macrophage metabolism to inflammation resolution and cardiac repair after MI. Repair macrophage metabolism is associated with both glycolytic and mitochondrial pathways of cellular metabolism. Glycolysis-derived lactate has the capacity to polarize macrophages to an antiinflammatory state. Fatty acids, including fatty acids derived from apoptotic cells, may enter the mitochondrion of the phagocyte (such as through MerTK-dependent efferocytosis) to fuel increases in mitochondrial respiration and the generation of NAD^+^. NAD^+^ can facilitate cellular signaling pathways that lead to induction of pro-repair cytokines. ARG, arginine; CPT, carnitine palmitoyltransferase; EPI, epigenetic; FA, fatty acid; FAO, fatty acid oxidation; GLN, glutamine; HIF, hypoxia-inducible factor; ITA, itaconate; α-KG, α-ketoglutarate; LA, lactylation; LIPA, lysosomal acid lipase; NAD^+^, nicotinamide adenine dinucleotide; NMN, nicotinamide mononucleotide; NFR2, nuclear factor erythroid 2–related factor 2; NR, nicotinamide riboside; PYR, pyruvate; PUT, putrescine; TCA, tricarboxylic acid.

Collectively, the metabolic signatures of antiinflammatory and pro-repair macrophages discussed above are consistent with the concept that specific metabolic pathways and metabolites have the capacity to enhance tissue repair through cardiac macrophages. A working model emerges from these data that outlines potential contributions of cardiac macrophage metabolism to cardiac healing (Figure 3).

## Future research directions and therapeutic implications

Like the overgeneralized dichotomy between M1 and M2 macrophages (137), current dogmas that divide inflammatory versus repair programming into glycolytic versus oxidative metabolism in the macrophage are also an oversimplification. Moreover, a profound limitation to our working models is that most of our immunometabolic insight originates from studies performed on cells cultured in vitro. New technical approaches are needed that preserve in vivo metabolic phenotypes. Another cautionary tale is that we too often make metabolic inferences from static quantifications of metabolite levels that can be deceiving. For example, higher metabolite concentrations can be a function of slower metabolite consumption or accelerated production. It is even formally possible that macrophages could undergo higher metabolite flux without any change in detectable levels. Looking ahead, experimental approaches that incorporate multiple orthogonal perspectives, including real-time in vivo metabolic tracing, will be key in understanding the true evolution of cardiac macrophage metabolism, as an element of macrophage function, after MI.

There is increasing evidence that mitochondrial dysfunction is associated with disease. Mitochondrial OXPHOS is a key signature of tissue macrophage homeostasis (138). Important questions remain unanswered with respect to the extent to which natural age-associated declines in macrophage metabolism may contribute to the efficiency of recovery after MI. Future studies are also needed to determine the extent to which metabolic MI risk factors contribute to cardiac macrophage responsiveness. For example, hyperlipidemic individuals are at increased risk for heart failure after MI (14), yet our understanding of how excess lipid load likely affects cardiac macrophage functions remains vague and incomplete. Also, advancements in the imaging of in vivo mitochondrial dynamics may provide further clues to the relationship between cardiac macrophage metabolism and disease progression (139).

Beyond the satisfaction of discovering fundamental new mechanisms linking metabolism to immune function, inflammation, and cardiac repair, this space also harbors powerful potential for therapeutic intervention. Macrophages have been implicated in the benefits associated with cell therapy after MI (140, 141). As such, optimizing cardiac macrophage metabolism to achieve optimal cardioprotection is a logical next step. Effective cardioprotection may be achieved through the administration of select bioactive metabolites such as itaconate or NAD^+^ (111), or alternatively by targeting of broader metabolic pathways, such as OXPHOS (142). Whatever the case may be, the field is moving fast, and new discoveries and therapeutic approaches are on the horizon.

## Version 1 09/15/2023

Electronic publication
